# Quinine Mixture

**Published:** 1898-02

**Authors:** 


**Quinine Mixture.**—The following is advantageous in irritable stomach when quinine is to be given:

℞ Quinine sulph.......................................gr. ii,

Ac. citrici............ ................................gr. vi,

Syr. simplic.........................................ʒ ss,

Syr. aurantii flor......... ..........................ʒ ss.

This is to be placed in a wine-glass containing bicarbonate of sodium (from three to five grains) in saturated solution, and drank during effervescence.—*Journal de Medical de Paris.*

Sally: "How I'd like to be one of them great actors or singers!"

Her Mother: "O! I dunno. It must be an unhealthy business."

Sally: "Why, ma?"

Her Mother: "Don't you allus see their names in the papers tellin' how they've been takin' patent medicines an' tonics an' sech?"—*Puck.*

